# Compound heterozygous splicing and missense variants in *MYO7A* in a Chinese patient with Usher syndrome

**DOI:** 10.3389/fmed.2026.1836745

**Published:** 2026-07-24

**Authors:** Juyi Li, Huihui Mao, Lu Li, Xiufang Wang, Guohua Yang, Jiguo Yu, Aiping Deng, Jifa Hu, Dan Wu, Peiyan Zhan, Yingbo Li

**Affiliations:** 1 Department of Pharmacy, Tongji Medical College, The Central Hospital of Wuhan, Huazhong University of Science and Technology, Wuhan, Hubei, China; 2 Department of Nephrology, Tongji Medical College, The Central Hospital of Wuhan, Huazhong University of Science and Technology, Wuhan, Hubei, China; 3 Department of Pain, Tongji Medical College, The Central Hospital of Wuhan, Huazhong University of Science and Technology, Wuhan, Hubei, China; 4 Department of Medical Genetics, School of Basic Medical Science, Wuhan University, Wuhan, Hubei, China; 5 Department of Ophthalmology, Tongji Medical College, The Central Hospital of Wuhan, Huazhong University of Science and Technology, Wuhan, Hubei, China; 6 Department of Teaching Office, Tongji Medical College, The Central Hospital of Wuhan, Huazhong University of Science and Technology, Wuhan, Hubei, China; 7 Department of Geriatrics, Tongji Medical College, The Central Hospital of Wuhan, Huazhong University of Science and Technology, Wuhan, Hubei, China

**Keywords:** genetics, minigene, *MYO7A*, Usher syndrome, whole-exome sequencing

## Abstract

**Objective:**

The objectives of the present study were to identify the genetic variations in a Chinese patient with Usher syndrome and to determine the pathogenicity of the identified variations.

**Methods:**

Whole-exome sequencing was performed for the proband. Alphafold3 and PyMOL software were used to determine the impact of a variation on three-dimensional protein structure. A Minigene Splicing Assay was performed to investigate the impact of a variant on *MYO7A* splicing.

**Results:**

Two compound heterozygous missense and splicing variations of *MYO7A* (NM_000260:c.487G > A:p.G163R, rs1472566324 and c.2187 + 2_2187 + 8del, rs1416744060) were identified in the proband. After glycine (G, WT) is replaced by arginine (R, rs1472566324), arginine forms additional hydrogen bonds with the surrounding amino acids. In the Minigene Splicing Assay, abnormal splicing bodies (associated with an Exon18 jump) were observed in the mutant plasmids (rs1416744060). This abnormal splicing event caused a deletion of 31 aa inside the protein, generating a truncated protein of 2,184 aa.

**Conclusion:**

Compound heterozygous missense and splicing variants of *MYO7A* (rs1472566324 and rs1416744060) were the likely pathogenic variants of a patient with Usher syndrome type 1B.

## 1 Introduction

Usher syndrome (USH) is an autosomal recessive genetic disorder characterized by partial or complete congenital sensorineural hearing loss (SNHL) and progressive vision loss due to retinitis pigmentosa (RP) (1). With a prevalence of 3–17 per 100,000 people, USH is the leading cause of congenital deafness and blindness, accounting for 50% of all cases (2). Most USH cases can be grouped into one of three subtypes: type I (USH1); type II (USH2); and type III (USH3). USH1, the most severe subtype of the disease, is characterized clinically by severe congenital bilateral SNHL, disappearance of vestibular response, and juvenile RP. USH2, the most common subtype, is characterized by mild to moderate congenital SNHL, normal vestibular response, and mid-term retinal pigment degeneration; USH3, the rarest subtype, is characterized by progressive congenital SNHL, vestibular response, and uncertain onset time of RP. As of 2026, an effective treatment for USH is not available. However, cataract surgery can help improve vision in around 50% of the patients with cataracts, hearing aids or cochlear implants can enhance patient hearing, and rehabilitation therapy can alleviate vestibular dysfunction (3).

To date, more than 15 genes have been implicated in USH pathogenesis, and the pathogenic mutations in these genes all exhibit autosomal recessive inheritance. Moreover, seven genes—*CDH23, CIB2*, *ESPN*, *MYO7A, PCDH15, USH1C,* and *USH1G*—have a demonstrated association with USH1; with *MYO7A* being the most common pathogenic gene, accounting for 29 to 50% of cases (2, 4). *MYO7A* on chromosome 11q13.5 encodes myosin VIIa protein, which is characteristically expressed in the retina and inner ear (5). Furthermore, dysfunction or deletion of *MYO7A*, which is associated with USH1B, has been shown to cause degeneration of sensory nerves in the retina and cochlea. With these observations in mind, we can conclude that an expansion in our understanding of the genetic epidemiology of USH assists disease diagnosis, and it may also inform targeted adjuvant therapy.

In the present study, we provide a detailed description of the phenotype and genetic characteristics of a *MYO7A*-related USH1 patient from China, and a preliminarily analysis of the associated pathogenic mechanism. These data are crucial for genetic counseling, eugenics, and disease monitoring and prediction, and they also provide key information for any researchers developing a gene editing therapy for USH.

## 2 Materials and methods

### 2.1 Patient

This study involved a Chinese Han family: the proband (male, 40 years old), his sister (38 years old) and his parents. The proband has congenital deafness and progressive retinitis pigmentosa, while the parents and sister are healthy. The proband underwent optical coherence tomography (OCT) and fundus examination (scanning laser ophthalmoscope, SLO) at Wuhan Central Hospital (China).

This study was approved by the ethics Committee of Wuhan Central Hospital. Written informed consent was obtained from all members of this family.

### 2.2 Next generation sequencing and variant analysis

A peripheral blood sample was obtained from each family member, and genomic DNA was then extracted from these samples. Exome libraries were prepared using the Novaseq X Plus-PE150 (Illumina) and SureSelect Human All Exon V6 (Agilent) kits. The hg19 human genome assembly (GRCH37) was used as a reference sequence for analyses. Candidate pathogenic variants were selected based on their gene frequency (<0.005) in the Genome Aggregation database^1^, the 1,000 Genomes Project database^2^, and the dbSNP database.^3^ The “harmfulness” of candidate pathogenic variants was predicted using SIFT, Polyphen-2, Mutation Taster, LRT, Mutation Assessor, and FATHMM. All variants were classified using the criteria of the American College of Medical Genetics and Genomics (6–8). From a consideration of the genetic history of the proband, compound heterozygous or homozygous variants were considered as candidate pathogenic variants. Finally, 15 candidate pathogenic genes for Usher syndrome were selected for further consideration (*ADGRV1*, *ARSG*, *CDH23*, *CIB2*, *CLRN1*, *ESPN*, *HARS1*, *MYO7A*, *PCDH15*, *PDZD7*, *PKDF125*, *USH1C*, *USH1G*, *USH2A*, *WHRN*). After candidate selection, sanger sequencing was used to validate candidate variations and recessive genetic patterns. The primers for Sanger sequencing were as follows: *MYO7A*-rs1416744060-F/R: GCTTCAGTTGCCATGTTCA/GAAGGAACCTAAAGATACAC; *MYO7A*-rs1472566324-F/R: AGGGCAAAAGACAAAGCAT/TTTCCGAAACGGCTTGAGT.

### 2.3 Molecular modeling

Molecular modeling was used to predict the influence of the p.G163R mutation on the three-dimensional structure of MYO7A protein. Structural models of wild type MYO7A and mutant proteins were constructed using AlphaFold3.^4^ These structures were then visualized using PyMOL.^5^ To analyze the surface electrostatic potential and hydrophobicity of these proteins, we used ChimeraX.^6^

### 2.4 Minigene construction and expression

To investigate the impact of the variant (*MYO7A*: c.2187 + 2_2187 + 8del) on mRNA splicing, a segment of intron 17 (337 bp), exon 18 (93 bp), and a segment of intron 18 (530 bp) of the *MYO7A* gene were cloned into the pcMINI minigene vector (Bioeagle, China). The wild-type and variant *MYO7A* gene segments were cloned separately to generate a wild-type pcMINI minigene construct and a mutant pcMINI minigene construct. In parallel, exon 17 (159 bp), intron 17 (457 bp), exon 18 (93 bp), and a segment of intron 18 (650 bp) of the *MYO7A* gene were cloned into the pcMINI-N minigene vector (Bioeagle, China). Again, the wild-type and variant *MYO7A* gene segments were cloned separately to generate a wild-type pcMINI-N minigene construct and a mutant pcMINI-N minigene construct. After their construction, the *MYO7A* wild-type minigene recombinant plasmids (pcMINI and pcMINI-N) and *MYO7A* mutant minigene recombinant plasmids (pcMINI and pcMINI-N) were transfected into HEK293T and HeLa cells, respectively. After subsequent incubation, total RNA was extracted from the transfected HEK293T/ HeLa cells. The total RNA was then reverse transcribed using the abovementioned kit. After end-point PCR, the reaction products were analyzed on a 2% agarose gel, and individual amplicons were recovered and sequenced. The primers used in this procedure are listed in Supplementary Table 1.

## 3 Results

### 3.1 Clinical examinations and pedigree analysis

**Figure 1:**
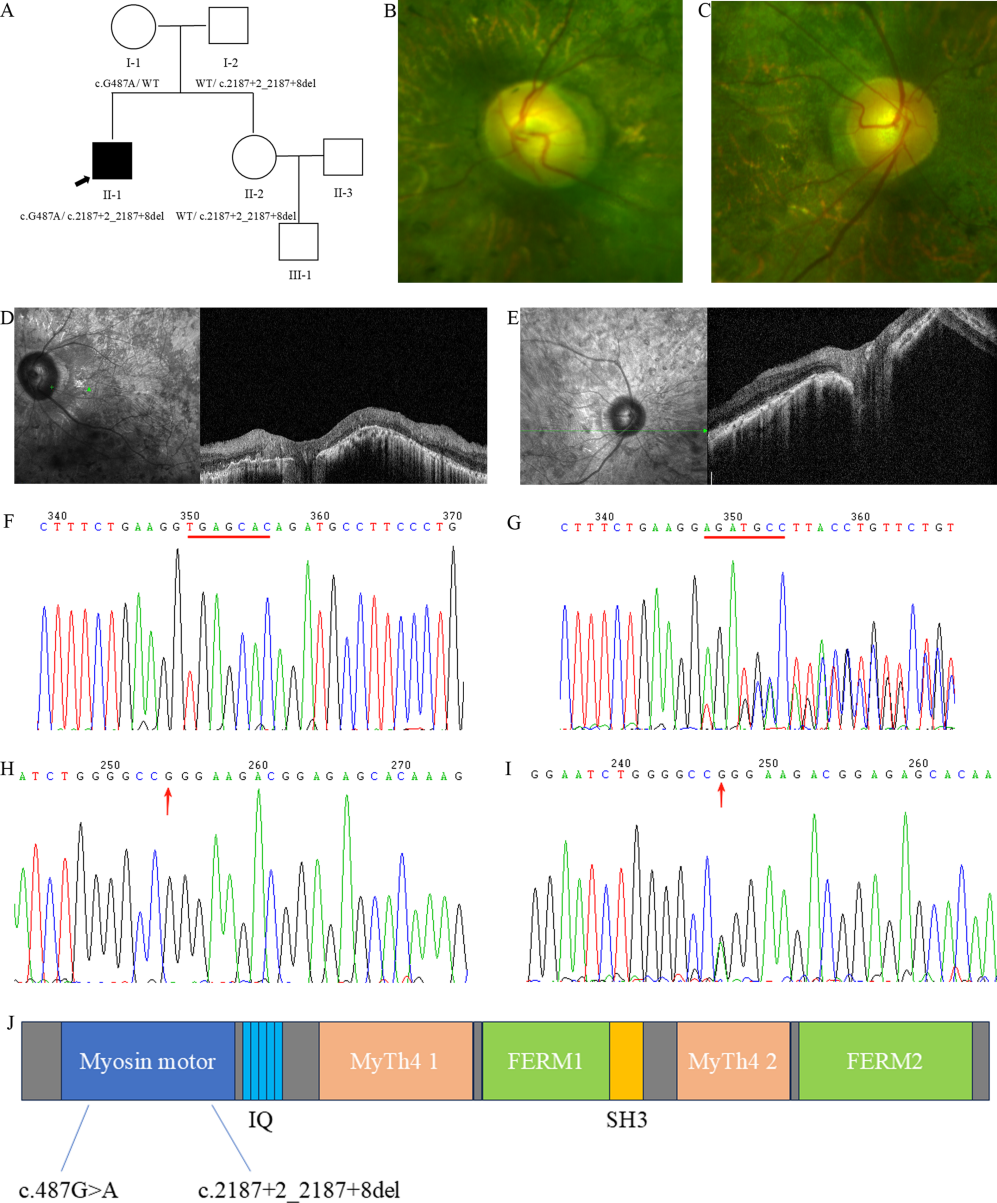
Clinical and basic characteristics of the family. **(A)** Pedigree and segregation analysis of disease-causing variants in *MYO7A*; **(B)** OCT imaging of proband’s left eye; **(C)** OCT imaging of proband’s right eye; **(D)** SLO imaging of proband’s left eye; **(E)** SLO imaging of proband’s right eye; **(F)** Wild type in *MYO7A*: c.2187 + 2_2187 + 8del (AAGGTGAGCACAGATG); **(G)** Mutant type in *MYO7A*: c.2187 + 2_2187 + 8del (AAGGAGATG); **(H)** Wild type in *MYO7A*:c.487G > A(GGGGCCGGGAA); **(I)** Mutant type in *MYO7A*: c.487G > A(GGGGCCAGGAA); **(J)** Myosin VIIA structural domains.

While family members were otherwise healthy, the 40-year-old male proband suffered from hearing loss, an inability to speak, and a gradual decline in vision (that started in childhood). An OCT examination revealed optic nerve and macular atrophy, thinning, and loss of outer layer structure (Figures 1B,C). A fundus examination (SLO) revealed wax yellow on the optic disc, thinning of arteries and veins, attenuation of blood vessels, bluish gray color on the retina, and deposition of bone cell like pigments (Figures 1D,E). Based on the ophthalmic examination results and known family history, our preliminarily diagnosis was that the proband has clinical manifestations of Usher syndrome, a recessive genetic disease (Figure 1A).

### 3.2 Genotyping and mutational analysis

To investigate the genetic basis of these signs, Whole-exome sequencing was performed on the proband (Supplementary Table 2); average sequencing depth on target, 211.84; bases covered by more than 50 sequencing reads, 91.0%. After consideration of the filtering criteria, recessive genetic patterns, and prioritized selection of 15 candidate genes, two compound heterozygous missense and splicing variations of *MYO7A* (NM_000260:c.487G > A:p.G163R, rs1472566324 and c.2187 + 2_2187 + 8del, rs1416744060) in the proband were associated with the disease. No other suspicious pathogenic variants were identified. The missense variant rs1472566324 had an extremely low frequency in GnomAD_exomes (A = 0.0000029) and TOPMED (0.000008), and was defined as pathogenic/ likely pathogenic in Clinvar. The splicing variation rs1416744060 also had an extremely low frequency in GnomAD_exomes (A = 0.0000007) and TOPMED (0.000008), and was also defined as pathogenic/ likely pathogenic in Clinvar. Sanger sequencing revealed that the proband had compound heterozygous variations of rs1472566324 and rs1416744060; the healthy father and sister were heterozygous for the variant rs1416744060, while the healthy mother was heterozygous for the variant rs1472566324 (Figures 1F–I). The positions of the MYO7A: c.487G > A and MYO7A: c.2187 + 22187 + 8del variants in the secondary structures of the MYO7A protein (Figure 1J). These results suggest that the compound heterozygous variants (c.487G>A and c.2187+2 2187+8del) co-segregate with clinical symptoms of the disease, and that carrying a single heterozygous pathogenic variant does not cause the disease.

### 3.3 Bioinformation analysis and structure modeling

**Figure 2:**
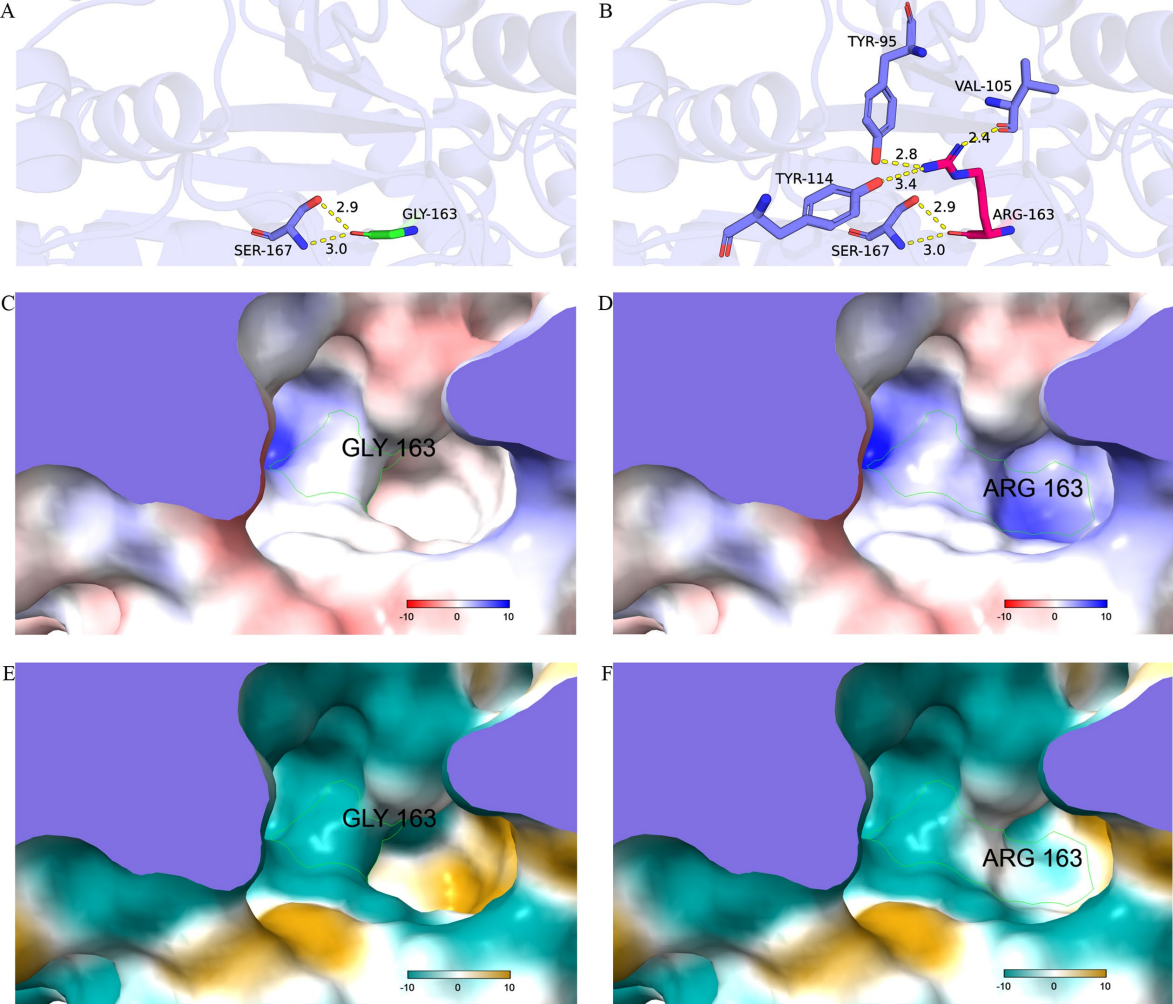
Molecular modeling the proteins of wild type and mutant type. **(A)** MYO7A c.487G>A: p.G163R wild-type glycine, with a single hydrogen atom side chain, does not participate in the formation of hydrogen bonds between side chains; **(B)** MYO7A c.487G>A: p.G163R mutant arginine is capable of establishing additional hydrogen bond connections with adjacent amino acid main chains or side chains (TYR 95, VAL 105, and TYR 114); The surface electrostatic potential of the proteins of wild type **(C)** and mutant type **(D)**; The hydrophilicity of the MYO7A proteins of wild type **(E)** and mutant type **(F)**.

Using *in silico* software, the *MYO7A* c.487G > A variant was predicted to be deleterious: SIFT (0.0, D); Polyphen2_HVAR (1.0, D); Polyphen2_HDIV (1.0, D); MutationTaster (1, D); LRT (0.000000, D); MutationAssessor (4.13, H); and FATHMM (−3.58, D). According to the FF, RDDC, and SpliceAI prediction websites, the *MYO7A* c.2187 + 2_2187 + 8del variant affected splicing. The *MYO7A* c.487G > A: p.G163R mutation changes a glycine residue to an arginine residue; notably, glycine residues have a single hydrogen atom for a side chain, and they cannot participate in hydrogen bonds between side chains in the wild-type protein (Figure 2A). In the MYO7A G163R mutant protein, a guanidine group at the end of the arginine side chain provides a strong hydrogen bond donor and acceptor, potentially serving as a new “anchor point” to establish additional hydrogen bond connections with adjacent amino acid main chains or side chains (TYR 95, VAL 105 and TYR 114) (Figure 2B). In an analysis of the surface electrostatic potential of the proteins (using ChimeraX software), we observed that the charge provided by glycine at position 163 is neutral in the wild-type protein, generating a neutral surface electrostatic potential (white coloring) in this region (Figure 2C). In the MYO7A G163R mutant protein, the positively charged arginine side chain is exposed on the protein surface, generating a positively charged patch (blue coloring) in this area, and completely altering the local electrostatic potential landscape (Figure 2D). Furthermore, structural model analysis (using ChimeraX software) revealed that, even though the mutated arginine is a typical hydrophilic amino acid, local hydrophobicity at position 163 is actually enhanced by the mutation (Figures 2E,F). A possible explanation is that the mutated arginine side chain is abnormally exposed to solvent, and the hydrophobic alkane portion of the side chain is buried inside the protein, packing against the hydrophobic core region.

### 3.4 *In vitro* splicing analysis of *MYO7A* c.2187 + 2_2187 + 8del by minigene assay

**Figure 3:**
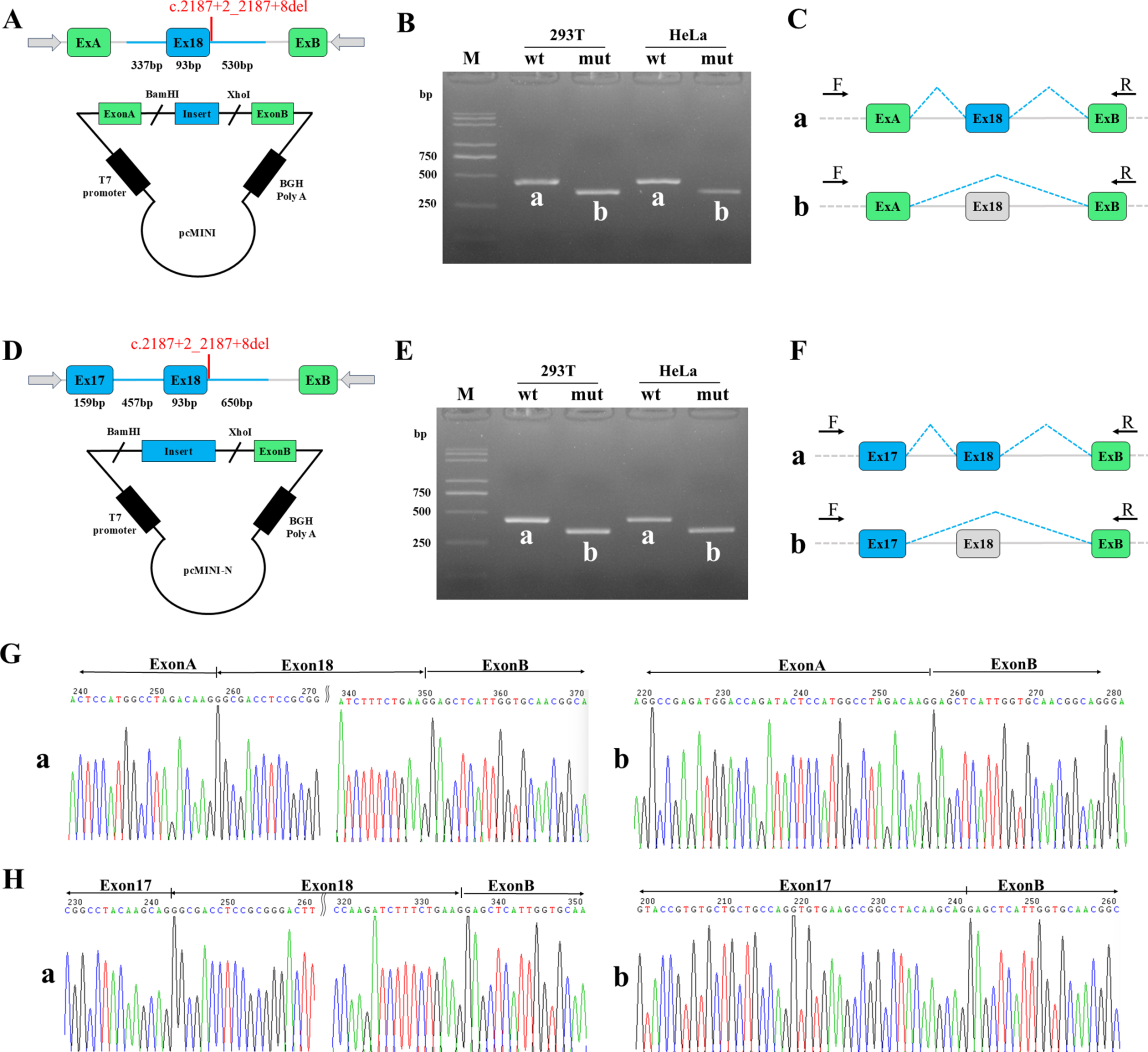
Minigene assay analysis of *MYO7A*: c.2187 + 2_2187 + 8del (pcMINI vector and pcMINI-N vector). **(A)** A segment of intron 17 (337 bp), exon 18 (93 bp), and a segment of intron 18 (530 bp) of the *MYO7A* gene were cloned into the pcMINI minigene vector; **(B)** DNA gel electrophoresis of the RT-PCR products in 293 T and Hela cells; bands from WT and the c.2187 + 2_2187 + 8del constructs are labeled as a and b, respectively; **(C)** Schematic diagram of cutting mode for bands **a** (B(a)) and **b** (B(b)); **(D)** Minigene insert was designed to include Exon 17 (159 bp), intron 17 (457 bp), exon 18 (93 bp), and a segment of intron 18 (650 bp) of the *MYO7A*; **(E)** Images of agarose gel electrophoresis of RT-PCR results for *MYO7A*, band a represents the WT and band b represents the c.2187 + 2_2187 + 8del; **(F)** Band **a** represents the cutting diagram of wild-type plasmids, while band **b** represents the cutting diagram of mutant plasmids; **(G)** Sanger sequencing results of excised bands for **a** (B(a, AAGGTGAGCACAGATG)) and **b** (B(b, AAGGAGATG)); **(H)** Band **a** represents the Sanger sequencing results for spliceosomes of wild-type plasmid (AAGGTGAGCACAGATG), while band **b** represents the Sanger sequencing results for spliceosomes of mutant plasmid (AAGGAGATG).

With the pcMINI vector (Figure 3A), RT-PCR analysis revealed that the wild-type construct produced a single band in HeLa and 293 T cells, consistent with the expected size (443 bp) of band a. Conversely, the mutant construct produced only band b in both HeLa and 293 T cells (Figure 3B). The wild-type and mutant bands produced in both cell lines were subsequently purified and analyzed directly by Sanger sequencing. The sequencing results revealed that wild-type band a was a normal cleavage band, with the cleavage mode being ExonA (192 bp) – Exon18 (93 bp) – ExonB (57 bp) (Figures 3Ca,Ga). In contrast, mutant band b was an abnormal cleavage band produced by Exon18 jumping, with the cleavage mode being ExonA (192 bp) – ExonB (57 bp) (Figures 3Cb,Gb).

To further validate the splicing pattern of this site, we used another plasmid pcMINI-N (Figure 3D). We also validated it in two types of cells (HeLa and 293 T cells), amplifying band a in the wild-type plasmid and band b in the mutant plasmid (Figure 3E). The sanger sequencing revealed the cleavage mode being Exon17 (159 bp) – Exon18 (93 bp) – ExonB (57 bp) (Figures 3Fa,Ha) in wild-type band a, in contrast, an abnormal cleavage pattern being Exon17 (159 bp) – ExonB (57 bp) (Figures 3Fb,Hb) was produced in mutant band b, with Exon18 jumping.

The results of our Minigene *in vitro* experiments provide evidence that the variant c.2187 + 2_2187 + 8del affects the normal splicing of *MYO7A* mRNA. In the mutant pcMINI and pcMINI-N vectors, an abnormal splicing event was observed, specifically Exon18 jumping. Further analysis of the sequence reveals that Exon18 jumps do not shift the reading frame; Exon18 jumps only produce a deletion of 31 aa inside the protein (c.2095_2187del p.Gly699_Lys729del), producing a truncated protein with a length of 2,184 aa.

## 4 Discussion

In the present study, we identified compound heterozygous missense and splicing variants of *MYO7A* (NM_000260:c.487G > A:p.G163R, rs1472566324 and c.2187 + 2_2187 + 8del, rs1416744060) in a 40-year-old male patient. The two variants co-segregated with the disorder in the wider family. While the rs1472566324 variant altered the electrostatic potential landscape, and hydrophilicity of the MYO7A protein, the rs1416744060 variant caused abnormal splicing. The results presented in this study provide evidence that the two compound heterozygous variants identified are the likely pathogenic origin of Usher syndrome type 1B in this family.

The human MYO7A (myosin VIIA) protein has 2,215 amino acids, and has multiple structural domains including: Myosin motor (65–741), five IQs (745–857), MyTH4 1 (1017–1,253), FERM 1 (1258–1,602), SH3 (1603–1,672), MyTH4 2 (1747–1896), and FERM 2 (1902–2,205) domains. The two variants identified in the present study (c.487G > A and c.2187 + 2_2187 + 8del in *MYO7A*) were located in the Myosin motor domain (“myosin head-like” domain), which is responsible for binding F-actin and ATP (9, 10). The proband investigated in this study had severe clinical symptoms, including hearing loss, an inability to speak, and a gradual decline in vision from childhood. In comparison, Li *et al* reported that patient G2-II1, who carried p. Leu728Pro (c.2183 T > C) and c.2187 + 2_ + 8 del variants in *MYO7A*, only exhibited nonsyndromic hearing loss (10). Although the above variants are all located in the motor domain, their clinical symptoms are greatly different. Hence, further in-depth research is needed to investigate the differences in clinical signs between the above two patients. Previous studies have found that mutations in the *MYO7A* gene are closely associated with both nonsyndromic hearing loss and USH1B (11, 12). However, *MYO7A*-related Usher syndrome typically presents as late-onset retinitis pigmentosa. We propose that further observation of the age of onset of eye disease in patients with nonsyndromic hearing loss is needed. In addition, recent studies have reported that some patients carrying pathogenic variants recorded in databases do not have clinical disease phenotypes. One possibility is that base mutations around the pathogenic variant site may weaken or offset the harm of the pathogenic variant (13). Hence, a comprehensive analysis of the bases around the pathogenic variant site is required to more accurately predict clinical characteristics.

Members of the myosin protein family are actin-based motor molecules with ATPase activity. The myosin VIIA protein is known to be especially important for intracellular transport. Myosin VIIA is characteristically expressed in the pigment epithelium, the photoreceptor cells of the retina, kidney, liver, testis, cochlea, and lymphocytes. In photoreceptor cells, myosin VIIA exists at the junction of cilia, regulating the transport of visual proteins. In retinal pigment epithelium cells, myosin VIIA is involved in the photoperiod-dependent transport of melanosomes, and it is required for the normal function of visual retinol circulation. In inner ear hair cells, myosin VIIA in complex with USH1C, USH1G, and CDH23 proteins mediates mechano-transduction in cochlear hair cells, a process that is required for normal hearing (9, 14, 15). Multiple studies have reported that homozygous mutations in *MYO7A* (including rs1472566324) and compound heterozygous mutations with other loci can cause nonsyndromic hearing loss or Usher syndrome (16–21). However, predictions of the harmfulness of these variants are often based on analyses with bioinformatics software, and the potential impacts of the resulting mutations on protein function are often ignored. In the present study, we propose that the MYO7A c.487G > A: p.G163R variant harbors additional hydrogen bonds between the introduced arginine and several other side chains (TYR 95, VAL 105 and TYR 114); this likely causes changes in protein stability and conformation. In addition, the mutation was shown to change the electrostatic potential and hydrophilicity of the mutant protein. Because this variant (*MYO7A*: c.487G > A:p.G163R, rs1472566324) introduces a mutation in the ATP binding site (158–165), it likely affects ATPase binding ability in myosin VIIA, potentially preventing ATP from being hydrolyzed into ADP, and ultimately rendering MYO7A G163R unable to provide power for intracellular movement. Collectively, the above findings provide a reference basis for future research on the pathogenic mechanism of these variants.

While the rs1416744060 variant has previously been suggested to affect gene splicing and thereby cause disease (10, 22, 23), functional experiments that demonstrate how this mutation affects splicing and causes disease have not been reported. In the present study, we confirm for the first time using MINIGENE functional experiments that the rs1416744060 variant causes an abnormal splicing event; thus, the rs1416744060 variant generated a truncated abnormal splicing transcript and reduced overall expression of the wild-type transcript.

In summary, over 500 *MYO7A* variants have been identified as potentially pathogenic for Usher syndrome to date, and we found that the variant (*MYO7A*: c.487G > A:p.G163R) may cause the loss of ATPase binding ability in myosin VIIA, potentially compromising energy utilization and the ability to participate in intracellular movement. The other variant (*MYO7A*: c.2187 + 2_2187 + 8del) caused abnormal splicing. The combined effects of the above two potentially pathogenic variants leads to the occurrence of Usher syndrome type 1B.

## Data Availability

The original contributions presented in the study are publicly available. This data can be found here: https://www.ncbi.nlm.nih.gov/bioproject/PRJNA1498697, PRJNA1498697.

## Footnotes

1
http://gnomAD.broadinstitute.org/

2
http://browser.1000genomes.org

3
http://www.ncbi.nlm.nih.gov/snp

4
https://www.alpha3manufacturing.com/

5
https://pymol.org/

6
https://www.cgl.ucsf.edu/chimerax/index.html
